# Distributions and Abundances of Sublineages of the N_2_-Fixing Cyanobacterium *Candidatus* Atelocyanobacterium thalassa (UCYN-A) in the New Caledonian Coral Lagoon

**DOI:** 10.3389/fmicb.2018.00554

**Published:** 2018-04-05

**Authors:** Britt A. Henke, Kendra A. Turk-Kubo, Sophie Bonnet, Jonathan P. Zehr

**Affiliations:** ^1^Department of Ocean Sciences, University of California, Santa Cruz, Santa Cruz, CA, United States; ^2^IRD, MIO, UM 110 – IRD Centre of Noumea, Aix-Marseille University, University of South Toulon Var, CNRS/INSU, Noumea, France

**Keywords:** *Candidatus* Atelocyanobacterium thalassa, nitrogen fixation, nitrogenase, *nifH*, oligotyping, UCYN-A, New Caledonia, Western Tropical South Pacific

## Abstract

Nitrogen (N_2_) fixation is a major source of nitrogen that supports primary production in the vast oligotrophic areas of the world’s oceans. The Western Tropical South Pacific has recently been identified as a hotspot for N_2_ fixation. In the Noumea lagoon (New Caledonia), high abundances of the unicellular N_2_-fixing cyanobacteria group A (UCYN-A), coupled with daytime N_2_ fixation rates associated with the <10 μm size fraction, suggest UCYN-A may be an important diazotroph (N_2_-fixer) in this region. However, little is known about the seasonal variability and diversity of UCYN-A there. To assess this, surface waters from a 12 km transect from the mouth of the Dumbea River to the Dumbea Pass were sampled monthly between July 2012 and March 2014. UCYN-A abundances for two of the defined sublineages, UCYN-A1 and UCYN-A2, were quantified using qPCR targeting the *nifH* gene, and the *nifH*-based diversity of UCYN-A was characterized by identifying oligotypes, alternative taxonomic units defined by nucleotide positions with high variability. UCYN-A abundances were dominated by the UCYN-A1 sublineage, peaked in September and October and could be predicted by a suite of nine environmental parameters. At the sublineage level, UCYN-A1 abundances could be predicted based on lower temperatures (<23°C), nitrate concentrations, precipitation, wind speed, while UCYN-A2 abundances could be predicted based on silica, and chlorophyll *a* concentrations, wind direction, precipitation, and wind speed. Using UCYN-A *nifH* oligotyping, similar environmental variables explained the relative abundances of sublineages and their associated oligotypes, with the notable exception of the UCYN-A2 oligotype (oligo43) which had relative abundance patterns distinct from the dominant UCYN-A2 oligotype (oligo3). The results support an emerging pattern that UCYN-A is comprised of a diverse group of strains, with sublineages that may have different ecological niches. By identifying environmental factors that influence the composition and abundance of UCYN-A sublineages, this study helps to explain global UCYN-A abundance patterns, and is important for understanding the significance of N_2_ fixation at local and global scales.

## Introduction

Biological nitrogen fixation is the conversion of dinitrogen (N_2_) gas into bioavailable nitrogen (N) by certain microorganisms (diazotrophs). This process can support up to 50% of the new production where primary production is N-limited (Karl et al., 1997) and thereby plays a fundamental role in the fixation of carbon and its export out of the photic zone (Karl et al., 2012). The Western Tropical South Pacific is a global hot spot of N_2_ fixation with rates averaging 570 μmol N m^-2^ d^-1^ in a vast area covering 5 × 10^12^ m^2^ (Bonnet et al., 2017). Oligotrophic coastal regions from the Western Tropical South Pacific can also be N-limited (Torréton et al., 2010), but little is known about the diazotroph communities there.

**FIGURE 1:**
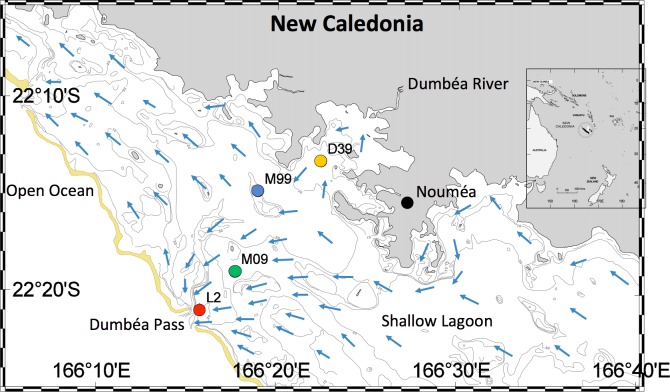
Map of study site, including stations D39, M99, M09, and L2. The New Caledonian (Noumea) lagoon is located in the Western Tropical South Pacific. Blue arrows show the hydrological circulation of the lagoon.

The Noumea lagoon is located off the southwest coast of New Caledonia in the Coral Sea of the Western Tropical South Pacific (**Figure 1**). Bounded by one of the world’s largest barrier reefs, the lagoon is a tropical low-nutrient low-chlorophyll system. It has an average depth of 17.5 m, along with many deeper canyons (∼60 m) and reef islands. The lagoon runs roughly 100 km from north to south and is 5 km wide in the north and 40 km wide in the south (Ouillon et al., 2010). The southeasterly trade winds drive the oligotrophic South Equatorial Current through the lagoon from the south to the north, with the Dumbea Pass being one of the main passages through which water exits in the northern part of the lagoon (**Figure 1**, Ouillon et al., 2010). Cooler sea and air temperatures prevail from June to September. October to December are generally warm and dry, while January to May are warm and wet (Ouillon et al., 2005, 2010). The Dumbea River provides a major freshwater input to the lagoon, especially during the wet months (Ouillon et al., 2005, 2010).

Primary production in the New Caledonian lagoon is N-limited (Torréton et al., 2010) providing a selective advantage for diazotrophs. High rates of N_2_ fixation have been measured in the large (>10 μm, filamentous diazotrophs) and small (<10 μm, unicellular pico- and nanoplanktonic bacteria and cyanobacteria) size-fractions in the lagoon (Garcia et al., 2007; Biegala and Raimbault, 2008; Bonnet et al., 2016). Large blooms of *Trichodesmium* spp., a colony-forming, filamentous diazotroph have been reported via satellite imagery (Dupouy et al., 2010; Ganachaud et al., 2010) and direct measurements (Renaud et al., 2005; Rodier and Le Borgne, 2008, 2010) and are correlated with warm sea surface temperatures (SST) (≥24°) (Rodier and Le Borgne, 2008), which are highest from November to May (Garcia et al., 2007; Dupouy et al., 2010; Ganachaud et al., 2010). The community composition of diazotrophs in the <10 μm size-fraction is less well-characterized. However, Biegala and Raimbault (2008), found unicellular picoplanktonic diazotrophs by using tyramide signal amplification fluorescence *in situ* hybridization (TSA-FISH) and a subsequent study confirmed the presence of the unicellular cyanobacterial group *Candidatus* Atelocyanobacterium thalassa (UCYN-A) in the lagoon (Turk-Kubo et al., 2015).

UCYN-A is a recently discovered (Zehr et al., 1998, 2001), uncultivated, diazotroph with widespread distribution (Cabello et al., 2015; Farnelid et al., 2016), that significantly contributes to N_2_ fixation in the oligotrophic open ocean (Montoya et al., 2004; Church et al., 2009; Martínez-Pérez et al., 2016). UCYN-A lives symbiotically with a single-celled eukaryotic prymnesiophyte (haptophyte) alga (Thompson et al., 2012). Nutrient exchange is believed to be the basis for the symbiosis; UCYN-A provides fixed nitrogen in return for fixed carbon (Thompson et al., 2012). The UCYN-A genome has been sequenced, showing that UCYN-A lacks oxygenic photosynthesis, carbon fixation and many other basic metabolic pathway genes (Tripp et al., 2010). This extreme genome reduction (Tripp et al., 2010; Bombar et al., 2014) and tight coupling of nutrient exchanges suggests an obligate symbiosis (Thompson et al., 2012).

Once thought to have low genetic diversity (Tripp et al., 2010), the UCYN-A lineage is now known to contain multiple sublineages. UCYN-A1 and UCYN-A2 are the dominant UCYN-A sublineages in terms of relative abundance (Turk-Kubo et al., 2017) and geographic range (Cabello et al., 2015; Farnelid et al., 2016). Several other sublineages have been reported, about which very little is known (Thompson et al., 2014; Turk-Kubo et al., 2017). UCYN-A1 and UCYN-A2 have genetically distinct prymnesiophyte hosts (Thompson et al., 2014). UCYN-A1 and UCYN-A2 also have morphological differences. UCYN-A1 and its host are much smaller (1.5 and 2.5 μm diameter, respectively) than UCYN-A2 and its host (4 and 8 μm diameter, respectively; Krupke et al., 2013; Cornejo-Castillo et al., 2016). A wide range of cell-specific N_2_-fixation rates have been measured (0.0074 fmol N cell^-1^ day^-1^ – 220 fmol N cell^-1^ day^-1^; Thompson et al., 2012; Krupke et al., 2013, 2015; and Martínez-Pérez et al., 2016), and it appears that the larger associations may have much higher cell-specific rates (Martínez-Pérez et al., 2016).

UCYN-A has been reported from the Noumea Lagoon in previous studies, but little is known about the biogeography and diversity of UCYN-A or the environmental factors that govern their distributions in this region. We hypothesized the abundance and diversity of UCYN-A in the Noumea Lagoon would vary seasonally, driven by changes in the environment (especially temperature). To address this hypothesis, we (1) quantified abundances and documented the spatial-temporal distributions of UCYN-A1 and UCYN-A2; (2) determined which environmental factors contributed most to overall abundances and distributions of UCYN-A; (3) differentiated the environmental factors associated with UCYN-A1 vs. UCYN-A2 abundances; (4) determined the diversity of UCYN-A in the lagoon both within and beyond the dominant sublineages; and (5) determined if there is a relationship between UCYN-A community composition (diversity) and specific environmental conditions.

## Materials and Methods

### Sampling

Using a trace metal clean Teflon pump connected to a polyethylene tube, surface seawater (3 m depth) was collected monthly between July 4, 2012 – April 3, 2014 in the lagoon along a transect running from the Dumbea Pass to the Dumbea Bay (**Figure 1**). The transect included four sampling sites. From the northeast to the southwest, the stations were: D39 (Dumbea Bay): 22°13.229′ S – 166°22.430′ E, M99: 22°14.798′ S – 166°18.954′ E, M09: 22°18.970′ S – 166°17.700′ E, and L2 (Dumbea Pass): 22°20.963′ S – 166°15.763′ E.

Samples for DNA analysis were immediately filtered through 25 mm diameter 0.2 μm pore-size Supor^®^ filters (Millipore, Billarica, CA, United States), using gentle peristaltic pumping. Sample volume varied (930–2350 ml) depending on the amount of water that could be filtered within a reasonable amount of time. All filters were stored at -80°C until shipment on dry ice from New Caledonia to the University of California, Santa Cruz.

### Temperature, Nutrient, and Chlorophyll Measurements

Surface temperature and fluorescence were recorded using a Seabird 911 plus conductivity-temperature-depth (CTD) profiler. Surface seawater samples (3 m depth) for nutrient analyses were collected in acid-washed polyethylene bottles, poisoned with HgCl_2_ (10 μg L^-1^ final concentration) and stored in the dark at 4°C until analysis.

Soluble reactive phosphorus (SRP) and NOx [nitrite (NO_2_^-^) + nitrate (NO_3_^-^)] concentrations were quantified using standard colorimetric techniques (Aminot and Kérouel, 2007) on a Bran Luebbe AA3 autoanalyzer. Detection limits for the procedures were 0.01 μM for SRP and 0.05 μM for NOx. The acidification protocol was used for chlorophyll *a* analysis (chl *a*). For this, seawater samples were collected in 0.55 L flasks, filtered onto GF/F Whatman filters, and extracted with 95% methanol after 30 min of incubation in the dark at ambient temperature (Herbland et al., 1985). Using a Turner Design fluorometer, the first fluorescence measurement was made, the sample was acidified (20 μl HCL 0.3 mol/l), and then the second measurement was made. The fluorometer used was outfitted with chl *a* extracted acidification module (module a 7200–040) calibrated with pure chl *a* standard (Sigma). For each sampling, a linear least squares regression was used to align the *in situ* CTD fluorescence measurements with the extracted chl *a* concentrations.

### Meteorological Data

Daily values were obtained for wind direction, wind velocity, and precipitation from the Météo-France station at Faubourg Blanchot (Noumea) 22°16.30′ S – 166°27.06′ E. These values were averaged to derive weekly average (mean of daily values the week prior to sampling) and monthly average (mean of daily values the month prior to sampling) covariate terms.

### Statistical Analysis of Environmental Data

A discriminant function analysis (DFA) model was developed using a quadratic model fitting routine to determine how environmental conditions varied across stations (e.g., if the station is predictable based on a set of environmental conditions). A forward stepwise DFA approach was used for model selection. Terms were added if they increased the overall discrimination ability without compromising the overall fit of the model, as evaluated using Pillai Trace estimation. All terms added to the model’s accuracy without compromising the model’s overall significance, so all terms were retained. This analysis utilized JMP^®^, Version *Pro 12*. SAS Institute Inc., Cary, NC, United States, 1989–2007.

### DNA Extraction

DNA was extracted using a Qiagen DNeasy Plant kit (Valencia, CA, United States), with protocol modifications optimized to recover high-quality DNA from cyanobacteria, including additional cell lysis steps of freeze-thaw cycles, agitation using a bead beater, as well as a proteinase K digestion (Moisander et al., 2008). The purity and quantity of DNA extracts was determined using a NanoDrop (Thermo Scientific, Waltham, MA, United States) according to the manufacturer’s guidelines.

### Determining UCYN-A Abundances Using Quantitative Polymerase Chain Reaction (qPCR)

UCYN-A was quantified using qPCR with Taqman^®^ assays. This study targeted UCYN-A1 (Church et al., 2005) and UCYN-A2 (Thompson et al., 2014). The UCYN-A2 assay is now known to also amplify the UCYN-A2, UCYN-A3, and UCYN-A4 sublineages (Farnelid et al., 2016). In keeping with the literature, all organisms targeted by the Thompson et al. (2014) UCYN-A2 primer are referred to as UCYN-A2 throughout this study even though UCYN-A3 and UCYN-A4 were also present. Preparation of standards, reaction volumes, thermocycling parameters, and standard curve-based abundance calculation are described in Goebel et al. (2010), with the exception of a 64^o^C annealing temperature for UCYN-A2.

The qPCR reaction efficiencies were 105 ± 3% for UCYN-A1 and 93 ± 3% for UCYN-A2. Based on the differences in sample volumes, the limits of detection (LOD) and quantification (LOQ) for all qPCR assays ranged between 17 and 145 *nifH* copies L^-1^, and between 133 and 1156 *nifH* copies L^-1^, respectively (Goebel et al., 2010). Samples were determined to be “detected, not quantified” (DNQ) when abundances were greater than the LOD, but less than the LOQ. *NifH* copies L^-1^ rather than cells L^-1^ is used as a proxy for abundance because the timing of replication, when cells have multiple *nifH* copies per genome, is unknown.

### UCYN-A Community Composition Determined by High-Throughput Sequencing of *nifH* Amplicons

Samples from each season were sequenced to evaluate if the UCYN-A sublineages targeted via qPCR represented all the UCYN-A phylotypes present in the lagoon, and to provide more detail about the seasonal patterns of the UCYN-A phylotypes. Twenty-eight lagoon samples (all four stations from the following dates: 1/31/13, 3/27/13, 4/3/14, 6/12/13, 8/13/13, 9/3/13, and 12/17/13) were chosen for analysis.

A nested PCR approach was used to amplify UCYN-A *nifH* from the lagoon samples, according to the method described in Turk-Kubo et al. (2017). UCYN-A *nifH* fragments were successfully amplified from all 28 samples. For each sample, triplicate PCR reactions were pooled. An additional 10 rounds of amplification to add sample-specific barcodes and sequencing adaptors was conducted at the DNA Service Facility at the University of Chicago, Illinois, prior to pooling and preparing for sequencing using the Illumina MiSeq platform.

From the 28 samples sequenced, 981,274 raw paired-end UCYN-A *nifH* reads were obtained. Sequences were processed according to a pipeline described in Turk-Kubo et al. (2017). This involved merging paired ends using Paired-End reAd mergeR (PEAR) software, quality filtering with Quantitative Insights Into Microbial Ecology (QIIME) scripts, as well as additional filtering in ARB to remove stop codons and non-*nifH* sequences. The majority of sequences (569,021 out of 981,274) passed quality filtering and were subsequently trimmed of primer regions, aligned to a reference alignment for UCYN-A sequences in a *nifH* database, and prepared for oligotyping analysis as per Turk-Kubo et al. (2017). Shannon entropy analysis and oligotyping was done using the oligotyping pipeline described Eren et al. (2013) and the same entropy positions and arguments described in Turk-Kubo et al. (2017). This analysis defined 60 unique oligotypes (alternative taxonomic groups) which represented 100.00% of the sequences submitted for analysis, with a purity score of 0.62. Raw sequence files are archived in the Sequence Read Archive (SRA) at the National Center for Biotechnology Information (NCBI) under BioSample Accession number PRJNA430445.

### Statistical Analysis of qPCR Data

#### Determinants of UCYN-A Abundance

To assess the relationships between potential predictor variables and total UCYN-A abundances (*nifH* copies L^-1^), a general linear modeling approach was used. Both categorical and continuous predictor variables were included. Categorical variables were station and sublineage (UCYN-A1 and UCYN-A2) while continuous predictor variables included temperature, chl *a*, NOx, SRP, weekly average wind direction, monthly average wind direction, weekly average wind speed, monthly average wind speed, weekly average precipitation, monthly average precipitation.

Variance inflation factor (VIF) scores for continuous variables were much less than 10 indicating the lack of co-linearity at a level that would compromise the modeling (Zuur et al., 2010). Starting with a full model, containing all terms and interactions, the reduced model was developed by sequentially dropping variables not contributing to model fit using Bayesian information criterion (BIC) standards. This analysis utilized JMP^®^, Version *Pro 12*. SAS Institute Inc., Cary, NC, United States, 1989–2007.

#### Environmental Characteristics That Correlate With UCYN-A1 and UCYN-A2 Abundances

To explore if the ratio of UCYN-A1 to UCYN-A2 could be predicted based on measured variables, a discriminant function analysis (DFA) was performed. Data was converted from continuous abundances to categorical states by distinguishing between two community states: (1) disproportionate numbers of UCYN-A1 or (2) disproportionate numbers of UCYN-A2. These two states were based on whether a sample ratio of UCYN-A1 to UCYN-A2 was greater than the long-term average value (Supplementary Table S3). A forward stepwise DFA approach was used for model selection. Terms were added if they increased the overall discrimination ability without compromising the overall fit of the model, evaluated using Pillai Trace estimation. This analysis utilized JMP^®^, Version *Pro 12*. SAS Institute Inc., Cary, NC, United States, 1989–2007.

### Statistical Analysis of Sequence Data

#### Spatial-Temporal Relationship Among UCYN-A Oligotype Distributions

A multidimensional scaling (MDS) plot based on Bray-Curtis (dis)similarities was constructed to determine which UCYN-A oligotypes (and sublineages) cluster together across samples. Using station and date as replicates, the resulting MDS plot is a graphical representation of relationships among oligotypes based on relative abundance. The obtained stress of the MDS (0.15) signified that it is a fairly good indicator of the relationship among oligotypes, as stress values range from 0 to 1.0, with zero signifying a perfect fit and all rank orders correctly represented by the relative distance between all pairs of points in the graph, and with values over 0.3 corresponding to near arbitrary placement of points in the graph (Clarke and Warwick, 2001). This MDS plot was constructed using the R package pyloseq (McMurdie and Holmes, 2013).

#### Relationship Between Predictor Variables and Oligotype Distributions

The relationship between corresponding oligotypes and the environmental patterns was assessed using a matrix-matching permutation-based test [BIO-ENV (Clarke and Warwick, 2001) method in PRIMER analytical software (vers. 7, PRIMER-E Ltd., Plymouth, United Kingdom)]. Two matrices were initially developed: (1) a similarity (or dis-similarity) matrix for pairwise comparisons of samples based on oligotype relative abundances and (2) a distance matrix for pairwise comparisons of samples based on environmental data (e.g., chlorophyll *a*). The biological similarity (relative abundance) was assessed using a Bray-Curtis approach and the environmental matrix was assessed using normalized data and Euclidean distances (Clarke and Ainsworth, 1993). In order to identify the subset of environmental properties with maximum correlation to the community pattern, the elements of the two corresponding similarity matrices were ranked, and the two matching sets of ranks were compared by calculating a Spearman correlation coefficient (ρ_s_). This was repeated after sequential generation of new environmental matrices that differed in the inclusion of environmental properties (e.g., all environmental properties except temperature, etc.). The value of an environmental property to the overall association of environmental and biotic matrices was assessed based on the change of fit to the overall model when the property was omitted from the generation of the environmental matrix. The significance level of the matches was calculated by comparing the value of ρ_s_ from any match to a null distribution of ρ_s_ values generated by iteratively (usually 1000 runs) randomizing the pairing of matrix elements in the Spearman Rank correlation (Clarke and Warwick, 2001).

Nonmetric multidimensional scaling (NMDS) plots were employed to create graphical representations of the relationship among samples based on the relative abundances of the various oligotypes and to illustrate temporal and spatial patterns of community structure. These nonmetric NMDS plots are just as described above; however, in this instance stations and dates were ordinated using oligotyes as replicates, whereas above the oligotypes were ordinated using stations and dates as replicates. Another difference is that nonmetric NMDS operates on the rank order of the elements in the similarity matrix, rather than on the matrix itself (as in the MDS plots described above), to construct a map of the samples in a specified number of dimensions.

## Results

### Environmental Conditions

**FIGURE 2:**
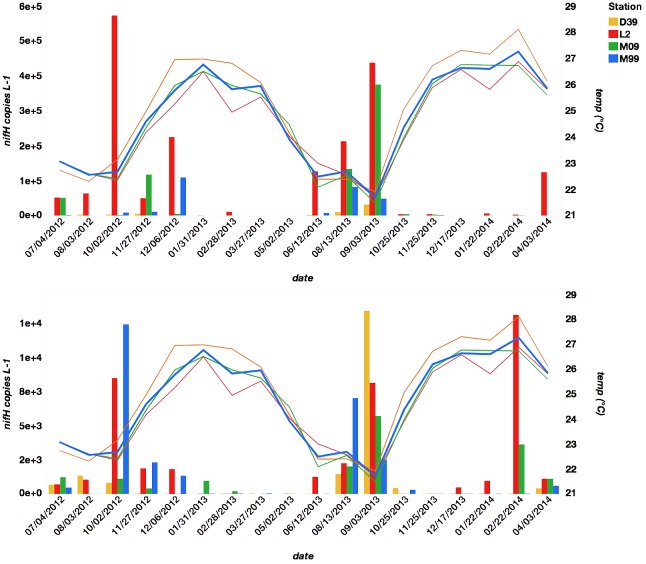
The relationship between UCYN-A1 *nifH* L^-1^ (top panel) and UCYN-A2 *nifH* L^-1^ (bottom panel) and temperature (°C) across date grouped by station.

Over the course of the study (July 4, 2012 – April 3, 2014) temperatures ranged from 21.5°C to 28.1°C, and were consistent across stations (± 0.71°C, **Figure 2**). Consistent with previous observations, the inner coastal station (D39) was generally slightly warmer than the outer station (L2), except in the austral winter when the trend briefly reversed (Ouillon et al., 2005). Peak temperatures were measured between the end of December and the end of February while minimum temperatures were between August and September. From November to May, mean monthly average wind direction ranged between northeast and southeast (45°–135°) with a consistently strong (≥9.5 m s^-1^) mean monthly wind speed. From June to October, mean monthly average wind speed was more southerly (>135°) with a weaker (<9.5 m s^-1^) mean monthly average wind speed. Colder SST occurred in conjunction with southern or southeastern wind. Chl *a* concentrations ranged from 0.13 μg L^-1^ to 0.99 μg L^-1^. NOx and SRP concentrations ranged from DL to 0.36 μM and DL to 0.37 μM, respectively. There was no discernable seasonal pattern associated with chl *a*, NOx, SRP, or with any other nutrient concentrations across stations. All environmental data is provided in Supplementary Table S1.

Environmental conditions at the stations reflected a gradient of environmental factors with station D39 being the most distinct based on DFA analysis (Supplementary Table S2). The top covariates that discriminate station D39 (positive values of scoring coefficient canonical axis 1) were ln(silicate) and ln(chl *a*), indicating the less oligotrophic nature of station D39. The DFA station model was accurate, correctly predicting station 96% of the time, and significant (using Pillai Trace estimation, *p* < 0.001).

### UCYN-A Abundances (qPCR)

**FIGURE 3:**
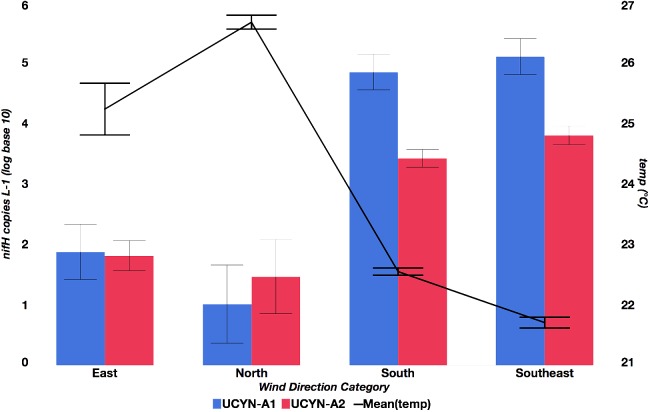
Relationship between mean UCYN-A1 and UCYN-A2 qPCR *nifH* L^-1^ (log base 10) and temperature across weekly average wind direction categories. Categories are defined as follows: north (340°–20°), east (80°–120°), southeast (121°–160°), and south (161°–110°).

Seasonal abundances of UCYN-A were variable (**Figure 2** and Supplementary Table S3). UCYN-A1 had higher mean qPCR-based abundances than UCYN-A2 (2.0 × 10^4^ ± 3.3 × 10^3^ *nifH* copies per L^-1^, including non-detects), but was detected sporadically (below the limit of detection in 18 out of 71 samples). UCYN-A2 mean abundances were an order of magnitude lower (2.0 × 10^3^ ± 3.4 × 10^2^ *nifH* copies per L^-1^, including non-detects), but UCYN-A2 was more consistently detected (only 5 samples below the limit of detection). Both UCYN-A1 and UCYN-A2 abundances peaked in the late austral winter (September and October), and were lower and more variable in the austral summer (December – May). Generally, the pattern of seasonal abundances varied inversely with annual temperature fluctuations (**Figure 2**) and were at their highest when wind was from the south or southeast (**Figure 3**). The high UCYN-A2 abundances in February of 2014 (10^4^ *nifH* copies per L^-1^) that coincided with high SST were an exception to the overall trend (**Figure 2**).

The ratio of UCYN-A1 to UCYN-A2 abundances (Supplementary Table S3) differed significantly across stations (*p* < 0.006). The ratio of UCYN-A1/UCYN-A2 was equivalent to the long-term average at stations M09 and M99 (11), but lower at station D39 (4.3) and higher at station L2 (19.2), indicating disproportionate numbers of UCYN-A2 at the costal station (D39) and disproportionate numbers of UCYN-A1 at the reef station (L2). Although UCYN-A2 abundances did not differ significantly across stations, maximum UCYN-A1 abundances were observed at the reef station (L2) and the difference in abundances across stations was significant (*p* < 0.001). UCYN-A1 was always detectable at station L2, where abundances ranged seasonally from 10^2^ – 10^5^ *nifH* copies L^-1^. Detection of UCYN-A1 was more sporadic at the inner stations.

### Determinants of UCYN-A Abundances

In order to determine which environmental factors can be used to predict UCYN-A abundances in this region, a reduced model was developed using the Bayesian information criteria (BIC) process. The reduced model was significant and explanatory (*p* < 0.0001, adjusted *r*^2^ of 0.57) and contained nine predictor variables, which were all individually significant (*p* < 0.05) or highly significant (*p* < 0.0001), suggesting the importance of a suite of factors in predicting abundances (**Table 1**).

**Table 1:** Linear Model to assess the relationship between predictor variables and nifH copies L^-1^ – Effect Test with Parameter Estimates.

Source	DF	Sum of squares	*F* Ratio	Prob > F	Parameter estimate
ln(temp)	1	53.492131	50.5696	<0.0001	-11.55497
Sublineage	1	20.143497	19.0430	<0.0001	
Weekly average precipitation	1	13.198302	12.4772	<0.0006	-0.106014
Monthly average wind speed	1	10.906539	10.3107	0.0018	0.3820217
Station	1	7.686324	7.2664	0.0082	
ln(chl *a*)	1	6.346722	6.0000	0.0160	-0.76317
ln(NOx)	1	6.952398	6.5726	0.0118	0.2530137
ln(temp)^∗^Sublineage	1	5.197246	4.9133	0.0289	
Sublineage^∗^Station	1	4.996625	4.7236	0.0321	

*P-values (Prob > F) indicate the fit of individual terms. The sign of parameter estimates indicates if the predictor variable is directly related (+) or inversely related (-) to nifH copies L^-1^. Parameter estimates for terms involving categorical variables are not shown.*

The most predictive term in the reduced model was ln(temp), and as indicated by the negative sign of the parameter estimate, the overall effect of temperature on UCYN-A abundance is negative (*p* < 0.0001, **Table 1**), after other variables have been modeled. The next most predictive terms in order were sublineage (UCYN-A1 or UCYN-A2), weekly average precipitation, monthly average wind speed, station, ln(NOx) and then ln(chl *a*). The last two terms in the model were interactions, sublineage^∗^ln(temp) and sublineage^∗^station. These last two terms indicate that depending on the sublineage (UCYN-A1 or UCYN-A2), temperature and station have different effects on abundances. Model parameter estimates indicate that overall UCYN-A abundance is negatively affected by increasing ln(chl *a*) concentrations and positively affected by ln(NOx) concentrations after all other factors have been accounted for.

Model findings are more predictive for UCYN-A1, because total UCYN-A1 abundances were 20x greater than total UCYN-A2 abundances. The significance of temperature in this model supports the high degree of variation in UCYN-A abundance over time (date). Temperature, however, is only one of nine predictor variables in this model, indicating that all nine factors are necessary to account for the total variation in UCYN-A abundance.

### Environmental Characteristics Associated With UCYN-A1 and UCYN-A2 Abundances

The results of the DFA of UCYN-A ratios indicated that the model was significant (*p* < 0.009) and the ratio of UCYN-A1 to UCYN-A2 could be predicted with high accuracy. The DFA model predicted 97% of events where the ratio of UCYN-A1 to UCYN-A2 was less than predicted and 81% of samples where the ratio was greater (Supplementary Table S3). Discrimination of UCYN-A2 from UCYN-A1 is driven by increasing ln(silicate), ln(temp), ln(chl *a*), monthly average wind speed, weekly average precipitation, and weekly average wind direction, in order of relative importance (**Table 2**). Discrimination of UCYN-A1 from UCYN-A2 is driven by increasing monthly precipitation average, ln(NOx), and weekly average wind speed, in order of relative importance.

**Table 2:** Discriminant function analysis (DFA) model to predict the ratio of UCYN-A1 to UCYN-A2 across samples – Scoring Coefficient and Canonical Structure Table.

Source	Scoring coefficients:	Total canonical
	Canon 1	structure: Canon 1
ln(temp)	5.7434101	0.6212042
ln(chl *a*)	0.4674547	0.482846
ln(silicate)	1.2997311	0.8002822
ln(NOx)	-0.321243	-0.267556
Monthly average precipitation	-0.34702	0.4116781
Monthly average wind speed	0.6159435	0.4301353
Weekly average precipitation	0.068581	0.2982829
Weekly average wind speed	-0.223516	0.2113514
Weekly average wind direction	0.0049191	0.02119

*Values indicate how environmental factors discriminate between two community states: (1) disproportionate numbers of UCYN-A1 or (2) disproportionate numbers of UCYN-A2. These two states were based on whether a sample ratio of UCYN-A1 to UCYN-A2 was greater than the long-term average value. Discrimination of UCYN-A2 from UCYN-A1 is associated with positive values of scoring coefficient canonical axis 1. Discrimination of UCYN-A1 from UCYN-A2 is associated with negative values of scoring coefficient canonical axis 1.*

Taken together, these models help explain the non-uniform distributions of UCYN-A1 and UCYN-A2 across stations, with a higher ratio of UCYN-A2 at the inner coastal station (D39) and higher abundances of UCYN-A1 at the outer reef station (L2). The results of this model reinforce findings from the overall abundance model, which is driven by UCYN-A1 abundances; ln(temp) and ln(chl *a*) are negative predictors of UCYN-A1 abundance, while ln(NOx) is a positive predictor of UCYN-A1 abundance.

### UCYN-A Genetic Diversity Within and Outside of Major Sublineages

To assess the genetic diversity of UCYN-A in the lagoon, oligotyping analysis was performed on amplified UCYN-A partial *nifH* gene sequences. The resulting UCYN-A *nifH* amplicon dataset had 44 previously identified oligotypes and 16 novel oligotypes, that represented all known sublineages of UCYN-A, as well as some potentially new sublineages. Twelve of these – in order of descending total relative abundance: oligo3, oligo1, oligo43, oligo4, oligo45, oligo46, oligo2, oligo40, oligo13, oligo30, oligo34, oligo27 – accounted for 96.7% of all sequences recovered (Supplementary Table S4). Four major oligotypes – oligo3, oligo1, oligo43, and oligo4 – accounted for 93.1% of all sequences recovered. The remainder of the dataset was comprised of minor oligotypes, present at low relative abundances across the dataset. Of the 16 newly defined oligotypes, several were in the UCYN-A3 sublineage and many others were in the UCYN-A2 sublineage. Other newly defined oligotypes were genetically distinct from previously defined oligotypes, and may be new sublineages. These are referred to here as UCYN-A7 and UCYN-A8, but more observations of these sequence types are needed to support these as new sublineages.

Oligo3 (UCYN-A2 sublineage; Turk-Kubo et al., 2017), dominated the UCYN-A *nifH* amplicon dataset, accounting for 45% of the total sequences. Oligo1 (UCYN-A1), was the second most abundant oligotype in this dataset, accounting for 31% of total recovered sequences. The third most abundant oligotype, oligo43 (UCYN-A2) made up 14% of total sequences. Thus far, oligo43 has been reported exclusively in the Noumea Lagoon of New Caledonia (Turk-Kubo et al., 2017). Oligo4, the fourth most abundant oligotype, clustered with the recently defined UCYN-A4 sublineage (Farnelid et al., 2016). Oligo4 accounted for 2% of total sequences. It is important to note that UCYN-A2 was preferentially amplified over UCYN-A1, as is indicated by the higher relative abundances of UCYN-A2 sequences, despite the reality that total UCYN-A1 qPCR abundances were 20x greater than those of UCYN-A2. Nevertheless, UCYN-A2 still comprises a low percentage of total UCYN-A sequences worldwide (Farnelid et al., 2016; Turk-Kubo et al., 2017), indicating that the UCYN-A2 bias observed in this study is not systematic.

### Spatial-Temporal Relationships Among Oligotype Distributions

**FIGURE 4:**
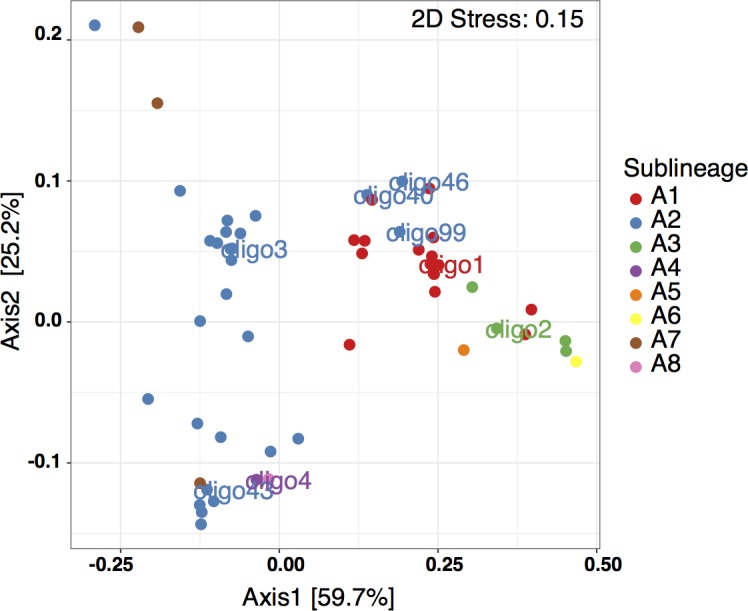
Metric Multidimensional Scaling (MDS) using the Bray–Curtis ecological index to determine dissimilarity between oligotypes based on relative abundance. Oligotypes are color-coded by sublineage.

In the MDS plot using Bray-Curtis based dissimilarities, oligotypes within the same sublineage largely co-occur (**Figure 4**). There are a few exceptions to this, with oligo46 (UCYN-A2), oligo40 (UCYN-A2) and oligo99 (UCYN-A2) clustering with the UCYN-A1 sublineage. Beyond these exceptions, there is a clear divide between the oligotypes associated with UCYN-A1 and those associated with UCYN-A2. Furthermore, UCYN-A4 (oligo4), UCYN-A7 and UCYN-A8 co-occur with UCYN-A2 oligotypes. In contrast, UCYN-A3 (oligo2), UCYN-A6 and UCYN-A5 sublineages cluster with UCYN-A1. These results indicate that specific assemblages of sublineages are found together in time and space.

### Relationship Between Predictor Variables and Oligotype Relative Abundances

**FIGURE 5:**
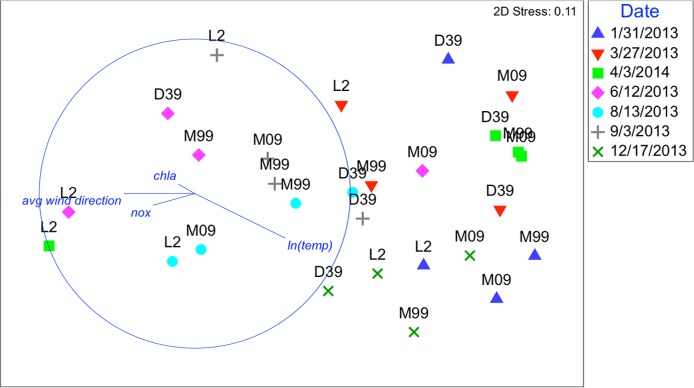
Nonmetric multidimensional scaling (NMDS): plot of 28 biological samples, coded by date and station with best explanatory environmental variable overlay. Data represent sequence relative abundances; resemblances based on Bray–Curtis similarity. Plots are overlaid with best explanatory environmental variable vectors, pointing in the direction of increasing ln(temp), chl *a*, NOx, and weekly average wind direction. The length of the vectors shows the strength of the relationship.

**FIGURE 6:**
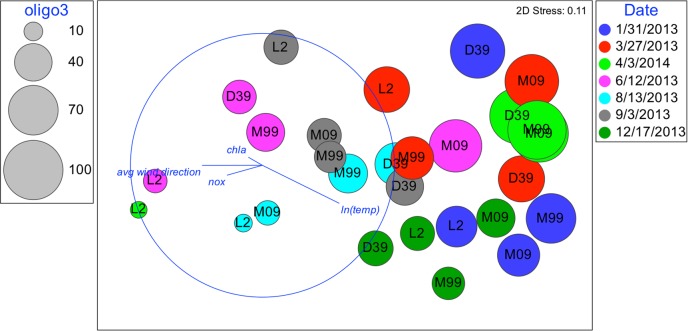
Nonmetric multidimensional scaling: plot of 28 biological samples, coded by date and station with best explanatory environmental variable overlay. Relative abundances of oligo3 are represented by the diameter of the circles.

**FIGURE 7:**
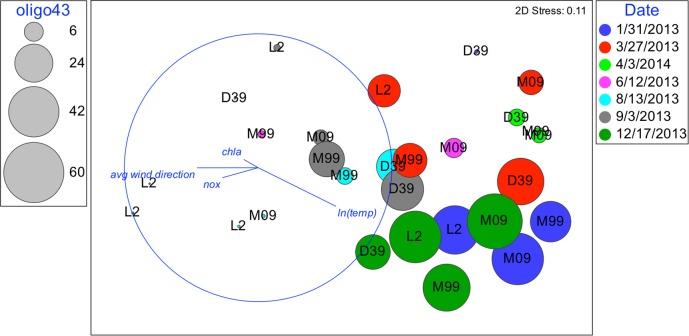
Nonmetric multidimensional scaling (NMDS): plot of 28 biological samples, coded by date and station with best explanatory environmental variable overlay. Relative abundances of oligo43 are represented by the diameter of the circles.

**FIGURE 8:**
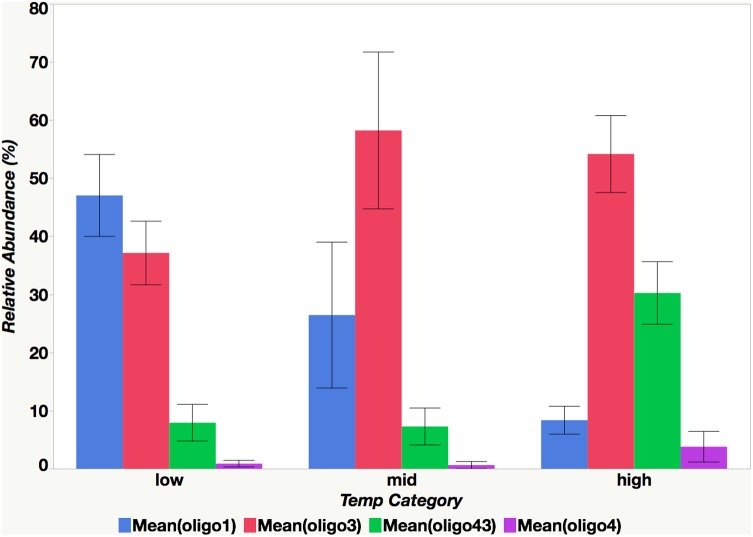
The relationship between mean oligotype relative abundance and temperature category. Categories are defined as follows: high (26°C ≤ x), mid (23°C ≤ x < 26°C), low (x < 23°C).

The BIO-ENV procedure showed that there was a strong correlation between oligotype community composition and environmental conditions (Spearman Rank correlation was 0.193 with a *p*-value of *p* < 0.022). The solution that maximized the Spearman rank correlation between the two resemblance matrices was a four-variable combination – ln(temp), chl *a*, NOx, and weekly average wind direction (ρ_s_ = 0.358, *p* < 0.001). As expected, these results support the qPCR-based models, with temperature [ln(temp)] and weekly average wind direction as strong predictors of oligotype composition (**Figures 5**–**8** and Supplementary Figures S1–S3). The co-occurrence of oligotypes from the same sublineage suggests that the same environmental characteristics select for all oligotypes within a sublineage.

Although oligotypes within a sublineage tended to respond similarly to environmental factors, there was an exception. Interestingly, oligo3 and oligo43, which both cluster with the UCYN-A2 sublineage, had somewhat different patterns of relative abundances (**Figures 6**, **7**). The relative abundance of oligo43 appears to correspond to ln(temp), as noted in the disproportionately large diameter of the circles in **Figure 7** associated with austral summer sampling dates (12/17/2013 and 1/31/2013) when SST is warmest. Of the three dates when temperatures are coldest (6/12/2013, 8/13/13, and 9/3/2013), oligo43 is virtually absent from the first two of these dates and only present at the inner stations on the third date. Using temperature categories [high (26°C ≤ x), mid (23°C ≤ x < 26°C), low (x < 23°C)], oligo3 did not have a consistent pattern with temperature, while oligo43 has higher relative abundances in the high temperature category (**Figure 8**). Oligo43 and oligo3 only differ genetically by one nucleotide, which indicates that distinct patterns of relative abundance may exist within sublineages.

## Discussion

### UCYN-A1 Is Present at Higher Abundances Than UCYN-A2 in the Noumea Lagoon

UCYN-A1 abundances reported in this study are in agreement with previous studies in the Western Tropical South Pacific around New Caledonia and Fiji, as well as in the Noumea lagoon (Moisander et al., 2010; Bonnet et al., 2015; Turk-Kubo et al., 2015; Stenegren et al., 2018). On an open-ocean transect in the Coral Sea from Australia to Fiji, maximum UCYN-A1 abundances (10^6^ *nifH* copies L^-1^) were measured at the stations nearest New Caledonia (Moisander et al., 2010). Similarly, Bonnet et al. (2015) sampled along a transect off the west coast of New Caledonia, extending southwest between 155°E and 165°E, and found UCYN-A1 abundances between 10^3^ and 10^5^ *nifH* copies L^-1^ with peak abundances off the coast of New Caledonia, just north of Dumbea Pass. In a *nifH* sequence analysis from the Coral Sea (off the northeast corner of Australia), UCYN-A1 comprised 42% of total *nifH* sequences (Messer et al., 2015). Stenegren et al. (2018) found patchy UCYN-A1 abundances that peaked around 10^4^ *nifH* copies L^-1^ around New Caledonia and south of Fiji (∼180°W). In a New Caledonian study that occurred south of this study location, UCYN-A1 abundances ranged from 10^3^ to 10^4^ *nifH* copies L^-1^ (Turk-Kubo et al., 2015).

There have been very few reports of UCYN-A2 in the Western Tropical South Pacific, and in several studies it is likely that the UCYN-A2 reported is actually UCYN-A3, as we now know that the Thompson et al. (2014) UCYN-A2 Taqman^®^ assay targets UCYN-A2, UCYN-A3 and UCYN-A4 sublineages (Farnelid et al., 2016). In the Noumea Lagoon, UCYN-A2 has been reported (and verified as UCYN-A2 via sequencing) at higher abundances than UCYN-A1 (10^4^ *nifH* copies L^-1^ and 10^3^ *nifH* copies L^-1^, respectively; Turk-Kubo et al., 2015). Bonnet et al. (2015) reported peak UCYN-A2 abundances of 10^3^ *nifH* copies L^-1^ off the southwest corner of New Caledonia, however, the sublineage present in that region is actually UCYN-A3 (Turk-Kubo et al., 2017). Stenegren et al. (2018) reported UCYN-A2 (10^4^ *nifH* copies L^-1^) around New Caledonia and Fiji (∼180°W), however, the Thompson et al. (2014) Taqman^®^ assay was used in this study, so it cannot be ruled out that they actually were detecting UCYN-A3, not UCYN-A2. In the northern part of the Coral Sea, Messer et al. (2015) identified low relative abundances of UCYN-A2 from *nifH* sequence data. Although quantitative UCYN-A2 data in the Western Tropical South Pacific is limited, existing data indicates that UCYN-A2 may be limited to coastal areas (around New Caledonia and the Fijian Islands), as well as in the Noumea lagoon.

### Total UCYN-A Abundances Are Negatively Correlated With Temperature

This is the first documentation of seasonal UCYN-A abundances in the New Caledonian lagoon with a September–October peak in abundances (10^4^ – 10^6^ *nifH* copies L^-1^) and lower, more-variable abundances from November to May. UCYN-A1 dictated this pattern of seasonal abundance, which fluctuated inversely with temperature. High austral spring abundances have been reported in previous studies [Biegala and Raimbault, 2008; Bonnet et al., 2015; and Messer et al., 2016 (high relative abundances)]. The pattern of UCYN-A abundance determined in this study is offset from that of *Trichodesmium*, which has maximum abundances from November to April (Garcia et al., 2007; Dupouy et al., 2010; Ganachaud et al., 2010). This temporal separation of niches contrasts with the spatial separation that has been observed in the open ocean, where *Trichodesmium* is often at higher abundances in surface waters, and UCYN-A often at higher abundances deeper in the water column (Moisander et al., 2010; Stenegren et al., 2018). These observations may be related to the warm temperature (≥∼25°C) generally thought to be required for *Trichodesmium* blooms (Capone et al., 1997; Montoya et al., 2004). The lagoon is N-limited throughout the seasonal cycle (Torréton et al., 2010), and these findings suggest that different diazotrophs are important to the ecology of the lagoon, depending on the season.

As indicated by the reduced model (**Table 1**), overall UCYN-A abundance is determined by nine environmental factors, including precipitation, wind speed, ln(NOx), and ln(chl *a*) concentrations. Temperature, which is often linked to cyanobacterial diazotroph distribution (Church et al., 2008; Moisander et al., 2010; Cabello et al., 2015), was the top predictor of UCYN-A abundance. Consistent with previous studies, which noted correspondence between high UCYN-A abundances and temperatures between 19°C and 24°C (Church et al., 2008; Langlois et al., 2008), a decline in UCYN-A abundances was observed in association with rising SST (x>24°C, **Figure 2**). Seasonal variation in irradiance, which is maximal from December to January (10 – 62 E m^2^ d^-1^) and minimal from June to July (5 – 25 E m^2^ d^-1^), contributes to seasonal temperature fluctuations (Torréton et al., 2010). Cooler SST may also be produced by shifts in wind direction (discussed below). Although it is not clear what other factors correlate with temperature, it does appear to be the primary control on UCYN-A abundance. Without the ability to characterize temperate optimums for this association in culture, however, it is difficult to establish a physiological link between temperature and growth, as other environmental variables might be responsible for the apparent temperature-abundance relationship.

### UCYN-A1 Abundances Are Correlated With South and Southeast Wind

A relationship between lower temperatures and southern or southeasterly wind was observed and could be accounted for by various explanations. Hénin and Cresswell (2005) found southeasterly wind events resulted in upwelling of cold water (<∼23°C) just north of Dumbea Pass. Southeastern wind also drives cool, oligotrophic South Equatorial Current water through the lagoon, augmenting the turn-over of lagoon water (**Figure 1**) (Torréton et al., 2010). Southerly wind and waves that may accompany this turnover are known to propagate through the reef passages (Ouillon et al., 2010). Conversely, northern or even eastern winds, to which maximum oligo3 relative abundances were correlated, would tend to have less of an effect on the lagoon community (Ouillon et al., 2010), especially at the more protected Dumbea Bay station (D39). Wind from the south and southeast are correlated with maximum UCYN-A1 abundances (by qPCR) and maximum oligo1 (UCYN-A1) relative abundances, which suggests that UCYN-A1 may be transported from the environment outside the lagoon. Previous UCYN-A studies support this distinction, finding higher UCYN-A2 to UCYN-A1 abundances in the lagoon (Turk-Kubo et al., 2015) and high abundances of UCYN-A1 outside the lagoon (Moisander et al., 2010; Bonnet et al., 2015).

### UCYN-A1 and UCYN-A2 Abundances Are Correlated With Distinct Environments

The DFA ratio model confirmed that UCYN-A1 and UCYN-A2 had different environmental predictors. UCYN-A2 abundances were positively affected by increasing temperature, silicate, and chl *a* concentrations, while UCYN-A1 abundances were inversely related to these factors. Wind speed was associated with abundances of both sublineages. Interestingly, SRP which has been speculated to control N_2_ fixation in the Western Tropical South Pacific (Van Den Broeck et al., 2004), was not correlated with abundances of either sublineage. The inverse relationship between UCYN-A1 abundance and temperature reported in this study is consistent with previous findings (Moisander et al., 2010; Bonnet et al., 2015). Messer et al. (2015) also identified the same temperature and silicate relationships in an Australian estuary, where higher UCYN-A2 abundance were found in warmer, higher silicate waters and higher UCYN-A1 abundances in colder, lower silicate waters (Messer et al., 2015). Messer et al. (2016) also reported a negative correlation between UCYN-A1 *nifH* relative abundances and silicate as well as phosphate in the Coral Sea, which was interpreted as an affinity of UCYN-A1 for oligotrophic conditions.

Other studies have identified correlations between UCYN-A abundances and environmental characteristics that appear to contradict those identified in this study. In a 23-day New Caledonian lagoon study with daily sampling, Turk-Kubo et al. (2015) found UCYN-A1 abundances positively correlated with chl *a* and UCYN-A2 abundances negatively correlated with temperature. The dissimilarity in study length and sampling frequency, and more narrow ranges of chl *a* (0.11 μg L^-1^ to 0.65 μg L^-1^) and temperature (25.30°C to 26.24°C) make comparisons challenging. However, UCYN-A2 abundances have been associated with colder waters in other studies including Bentzon-Tilia et al. (2015) and Turk-Kubo et al. (2017), which identified a predominance of UCYN-A2 sequences in the cold Danish Strait waters. Clearly, the factors driving UCYN-A abundances are complex and often intertwined. Further research will be needed to assess the correlations identified in this study, to see which factors continue to prove useful predictors of UCYN-A abundances on a broader scale. The higher taxonomic resolution afforded by oligotyping may be an important tool for understanding global patterns of UCYN-A distributions.

### UCYN-A Genetic Diversity Is Predictable From Environmental Characteristics

The oligotyping method (Eren et al., 2013) is an emerging tool that helps to characterize UCYN-A community composition and interpret geographic distribution of sublineages (Turk-Kubo et al., 2017). 44 UCYN-A oligotypes had been defined using a global survey of UCYN-A sequences that also included UCYN-A sequences from previous studies (Bentzon-Tilia et al., 2015; Messer et al., 2015; Turk-Kubo et al., 2015; Messer et al., 2016). Turk-Kubo et al. (2017) identified four dominant oligotypes: oligo1(UCYN-A1), oligo2 (UCYN-A3), oligo3 (UCYN-A2) and oligo4 (UCYN-A4). Three of these oligotypes [oligo1 (UCYN-A1), oligo3 (UCYN-A2), and oligo4 (UCYN-A4)] accounted for 78% of the sequences recovered in this study, which underscores that these oligotypes are widely distributed. The characterization of 16 new oligotypes, and possibly new sublineages (if these clusters are confirmed in subsequent observations), likely indicates that there is UCYN-A diversity yet to be discovered. Findings may also be an indication of the high endemism known in New Caledonia (Morat, 1993; Veillon et al., 2013).

As indicated by the co-occurrence of oligotypes from the same sublineage, the point mutations and subsequent genetic variability that may give rise to distinct oligotypes may not be ecologically significant. Consistent with the findings of the ratio of UCYN-A1 to UCYN-A2 DFA, the relative abundances of oligo1 (UCYN-A1) correlate with lower temperature (<23°C) and moderate NOx concentrations (0.05 μM –0.075 μM) while oligo43 (UCYN-A2) relative abundances correlate with higher temperatures (≥26°C).

Although oligotypes within sublineages tend to be found together, there are some notable exceptions. For instance, relative abundances of oligo3 (UCYN-A2) and oligo43 (UCYN-A2) differ with respect to SST. Oligo3 relative abundances did not have a consistent relationship with temperature, and this is in contrast to oligo43 relative abundances, which correlated with high temperatures (≥26°C). Diversity at the oligotype level of resolution may also help explain the discrepancy between the results of this study, which found that UCYN-A2 abundances correlate with higher temperatures, despite the fact that UCYN-A2 is the dominant sublineage in the cold Danish Strait waters (Turk-Kubo et al., 2017). Oligo43 has only been identified in the Noumea lagoon. Conversely, oligo3 dominated Danish Strait samples. These findings strongly suggest that fine-scale sequence variation may be important in understanding the global distributions of UCYN-A populations and that environmental predictors are not always consistent for all oligotypes within the same sublineage.

### Co-occurrence Patterns May Indicate Distinct UCYN-A1 and UCYN-A2 Niches

Results of both the qualitative and quantitative aspects of this study indicate that sublineage-level distinctions are valuable and support the emerging concept that UCYN-A1 and UCYN-A2 are distinct ecotypes. Since these sublineages differ in growth (Turk-Kubo et al., 2015; Martínez-Pérez et al., 2016) and N_2_ fixation rates (Martínez-Pérez et al., 2016), accurate predictions of distributions, timing, and abundances of UCYN-A1 and UCYN-A2 are necessary for modeling oceanic N_2_ fixation. Previous studies also indicated that sublineages may have different patterns of abundances, with the UCYN-A1/prymesiophyte association having higher abundances in open ocean waters (Thompson et al., 2014; Farnelid et al., 2016) and the UCYN-A2/prymnesiophyte association possibly more commonly found at high abundances in coastal regions (Thompson et al., 2014; Farnelid et al., 2016). Additionally, the ordination of oligotypes by Bray-Curtis (dis)similarities observed in this study is in agreement with the pattern observed in Turk-Kubo et al. (2017) who found this difference to be driven by the co-occurrence of the UCYN-A1 and UCYN-A3 in oligotrophic samples, which contrasts with the appearance of UCYN-A2 and UCYN-A4 in coastal samples. As observed in this study, however, UCYN-A1 and UCYN-A2 are also known to co-occur, especially in coastal regions influenced by oligotrophic water. Although further characterization of the biology underlying these two symbiotic associations is needed to verify that they are distinct ecotypes, this study provides evidence to support the emerging pattern that different sublineages have distinct ecological niches.

## Conclusion

The results of this study are consistent with previous reports that UCYN-A is found in colder waters (19°C – 24°C) than were previously associated with diazotrophs (Moisander et al., 2010). UCYN-A has been reported from higher latitudes (Moisander et al., 2010), coastal systems (Bentzon-Tilia et al., 2015) hypersaline (Messer et al., 2015), and N-replete waters (Short and Zehr, 2007; Moisander et al., 2010; Mulholland et al., 2012). UCYN-A has a significant N_2_ fixation capacity (>100 fmol N cell^-1^ day^-1^) that is equal to that of *Trichodesmium* (Martínez-Pérez et al., 2016), and with growth rates five to ten times higher than those of *Trichodesmium* (Martínez-Pérez et al., 2016). Thus, our improved understanding of UCYN-A is challenging everything we know about diazotrophs, including the long-held paradigm that *Trichodesmium* and diatom symbionts are largely responsible for the majority of marine N_2_ fixation (Villareal and Carpenter, 1989; Villareal, 1990; Capone, 1997). Continued investigation of UCYN-A distribution, abundance, and associated N_2_ fixation rates are thus important for understanding global marine biogeochemical cycles.

The late austral winter peak in UCYN-A abundances documented in this study corresponds with previous reports of high day-time N_2_ fixation rates in the small (<10 μm, pico- and nanoplankton) size-fractions from August to October (Garcia et al., 2007; Biegala and Raimbault, 2008; Bonnet et al., 2015). With a few exceptions (Garcia et al., 2007; Biegala and Raimbault, 2008; Bonnet et al., 2015), previous N_2_ fixation studies from the New Caledonian lagoon and Western Tropical South Pacific are largely focused on the austral summer, which has been considered the main N_2_ fixation season in the region (Garcia et al., 2007). Nonetheless, moderate pico- and nanoplankton N_2_ fixation rates (0.4–0.7 nmol N l^-2^ d^-1^) have been documented in the lagoon (Biegala and Raimbault, 2008) and around New Caledonia (Bonnet et al., 2015) in the austral spring, at times with 78% of biological nitrogen fixation occurring during the day-time (Garcia et al., 2007). Taken together, the literature suggests that “off-season” N_2_ fixation may be an important source of new N in the Noumea lagoon, and in the context of these reports, the high austral spring UCYN-A abundances reported in this study, and day-time N_2_-fixation, which is consistent with the *nifH* expression patterns of UCYN-A (Church et al., 2005), suggest that UCYN-A may be a significant contributor to “off-season” N_2_ fixation in the Western Tropical South Pacific. As primary production peaks in the austral winter (Lyne et al., 2005; Brewer et al., 2007), this source of added N could have a large effect on primary production, especially when coupled with other N sources.

Results from this study underscore the need for both qPCR and sequence analysis targeting the *nifH* gene for a comprehensive assessment of UCYN-A. Quantitative analysis (qPCR) is essential for quantifying abundances, but is limited due to the difficulty of designing primers to distinguish between the different subclades along this relatively short region of the *nifH* gene. Sequences are necessary to differentiate between UCYN-A subclades.

## Author Contributions

KT-K and JZ designed the experiment. SB collected the samples. BH preformed the experiments and analyzed the data, including statistical analysis. BH wrote the paper with contributions from KT-K, BS, and JZ.

## Conflict of Interest Statement

The authors declare that the research was conducted in the absence of any commercial or financial relationships that could be construed as a potential conflict of interest.
