# Supplementary material for: Distributions and Abundances of Sublineages of the N2-Fixing Cyanobacterium Candidatus Atelocyanobacterium thalassa (UCYN-A) in the New Caledonian Coral Lagoon
Source: Front Microbiol. 2018 Apr 5;9:554. doi: 10.3389/fmicb.2018.00554 (PMC5895702; doi:10.3389/fmicb.2018.00554)
Supplement: Supplementary file 1 [file Table_1.PDF]

Supplementary Table 1: Summary of environmental data. Meterological data is from the Météo-France station at Faubourg Blanchot (Nouméa).

Sample	Station	Date	Temp (°C)	Chl *a* (µg L^{-1})	Silicate (µM)	Nox (µM)	SRP (µM)	Monthly ave. precipitation (mm)	Monthly ave. wind speed (M/S)	Monthly ave. wind direction (°)
64813	L2	7/4/12	23.05	0.25	0.95	0.17	0.03	1.36	7.68	141.03
64814	M09	7/4/12	23.08	0.24	1.02	0.03	0.1	1.36	7.68	141.03
64815	M99	7/4/12	23.06	0.23	1.54	0.03	0.02	1.36	7.68	141.03
64816	D39	7/4/12	22.73	0.42	7.32	0.1	0.02	1.36	7.68	141.03
64817	L2	8/3/12	22.6	0.39	1.22	0.05	0.05	1.94	7.16	159.03
64818	M09	8/3/12	22.6	0.34	1.11	0.03	0.03	1.94	7.16	159.03
64819	M99	8/3/12	22.56	0.3	1.64	0.01	0.04	1.94	7.16	159.03
64820	D39	8/3/12	22.31	0.35	5.53	0.07	0.05	1.94	7.16	159.03
64821	L2	10/2/12	22.35	0.22	1.28	0.13	0.02	1.85	9.47	130.97
64822	M09	10/2/12	22.41	0.19	1.62	0.11	0.2	1.85	9.47	130.97
64823	M99	10/2/12	22.67	0.22	2.31	0.05	0.37	1.85	9.47	130.97
64824	D39	10/2/12	23.14	0.31	2.73	0.1	0.09	1.85	9.47	130.97
64825	L2	11/27/12	24.16	0.38	0.84	0.02	0.07	1.53	9.49	122.33
64826	M09	11/27/12	24.35	0.21	0.98	0.06	0.04	1.53	9.49	122.33
64827	M99	11/27/12	24.59	0.22	2.2	0.03	0.1	1.53	9.49	122.33
64828	D39	11/27/12	24.97	0.16	3.15	0.02	0.06	1.53	9.49	122.33
64829	L2	12/6/12	25.24	0.17	0.99	0.22	0.2	2.62	9.14	118.28
64830	M09	12/6/12	25.95	0	1.54	0.12	0.05	2.62	9.14	118.28
64831	M99	12/6/12	25.76	0	1.17	0.17	0.05	2.62	9.14	118.28
64832	D39	12/6/12	26.94	0.31	7.95	0.01	0.16	2.62	9.14	118.28
64833	L2	1/31/13	26.47	0.22	1.48	0.12	0.06	0.34	na	na
64834	M09	1/31/13	26.49	0.19	1.77	0.03	0.05	0.34	na	na
64835	M99	1/31/13	26.75	0.22	3.34	0.02	0.09	0.34	na	na

64836	D39	1/31/13	26.96	0.31	5.79	0.04	0.03	0.34	na	na
64837	L2	2/28/13	24.93	0.31	1.64	0.05	0.07	0.19	na	na
64838	M09	2/28/13	25.95	0.35	2.35	0.02	0.05	0.19	na	na
64839	M99	2/28/13	25.81	0.46	3.24	0.02	0.04	0.19	na	na
64840	D39	2/28/13	26.8	0.67	4.6	0.02	0.04	0.19	na	na
64841	L2	3/27/13	25.5	0.38	2.24	0.06	0.02	4.74	9.12	110
64842	M09	3/27/13	25.63	0.28	2.03	0.02	0.03	4.74	9.12	110
64843	M99	3/27/13	25.94	0.35	3.51	0.03	0.01	4.74	9.12	110
64844	D39	3/27/13	26.07	0.52	9.36	0.03	0.02	4.74	9.12	110
64845	L2	5/2/13	24.03	0.41	2.09	0.13	0.14	1.87	7.62	149.35
64846	M09	5/2/13	24.48	0.32	4.3	0.05	0.01	1.87	7.62	149.35
64847	M99	5/2/13	23.9	0.38	3.96	0.07	0.02	1.87	7.62	149.35
64848	D39	5/2/13	24.16	0.99	15.01	0.11	0.05	1.87	7.62	149.35
64849	L2	6/12/13	23	0.24	1.93	0.36	0.13	2.3	7.92	142.33
64850	M09	6/12/13	22.08	0.25	2.01	0	0.07	2.3	7.92	142.33
64851	M99	6/12/13	22.49	0.34	2.93	0.07	0.02	2.3	7.92	142.33
64852	D39	6/12/13	22.39	0.5	5.23	0.03	0.03	2.3	7.92	142.33
64853	L2	8/13/13	22.56	0.23	1.78	0.07	0.04	1.82	7.56	144.19
64854	M09	8/13/13	22.54	0.26	2.01	0.07	0.04	1.82	7.56	144.19
64855	M99	8/13/13	22.69	0.16	1.99	0.04	0.03	1.82	7.56	144.19
64856	D39	8/13/13	22.4	0.37	4.52	0.02	0.03	1.82	7.56	144.19
64857	L2	9/3/13	21.71	0.31	1.54	0.04	0.14	1.49	7.59	165.67
64858	M09	9/3/13	21.48	0.26	1.95	0.04	0.03	1.49	7.59	165.67
64859	M99	9/3/13	21.71	0.21	2.92	0.05	0.27	1.49	7.59	165.67
64860	D39	9/3/13	21.92	0.25	5.13	0.02	0.02	1.49	7.59	165.67
64861	L2	10/25/13	23.88	0.33	1.85	0.08	0.04	1.87	9.2	141.61
64862	M09	10/25/13	23.97	0.5	1.85	0.06	0.03	1.87	9.2	141.61
64863	M99	10/25/13	24.37	0.29	3.56	0.13	0.02	1.87	9.2	141.61
64864	D39	10/25/13	25.06	0.54	7.61	0.06	0.01	1.87	9.2	141.61
64865	L2	11/25/13	25.87	0.13	1.81	0.06	0.03	1.63	8.24	153
64866	M09	11/25/13	26.02	0.25	2.12	0.09	0.02	1.63	8.24	153

64867	M99	11/25/13	26.18	0.23	2.13	0.15	0.01	1.63	8.24	153
64868	D39	11/25/13	26.71	0.67	2.97	0.05	0.01	1.63	8.24	153
64869	L2	12/17/13	26.55	0.31	1.88	0.06	0	2.52	8.75	123.87
64870	M09	12/17/13	26.75	0.34	2.3	0.08	0.01	2.52	8.75	123.87
64871	M99	12/17/13	26.62	0.26	2.95	0.06	0.01	2.52	8.75	123.87
64872	D39	12/17/13	27.29	0.38	3.96	0.07	0.02	2.52	8.75	123.87
64873	L2	1/22/14	25.8	0.3	1.69	0.12	0.02	5.09	11.26	121.94
64875	M99	1/22/14	26.58	0.36	10.12	0.08	0.02	5.09	11.26	121.94
64876	D39	1/22/14	27.14	0.56	6.83	0.11	0.03	5.09	11.26	121.94
64877	L2	2/22/14	26.86	0.15	2.92	0	0.02	4.89	8.66	137
64878	M09	2/22/14	26.71	0.22	2.91	0	0.02	4.89	8.66	137
64879	M99	2/22/14	27.24	0.22	3.5	0.06	0.01	4.89	8.66	137
64880	D39	2/22/14	28.08	0.35	5.39	0	0	4.89	8.66	137
64881	L2	4/3/14	25.81	0	2.79	0.02	0.03	0.24	7.73	123
64882	M09	4/3/14	25.6	0	3.36	0.02	0.01	0.24	7.73	123
64883	M99	4/3/14	25.86	0	4.18	0.05	0	0.24	7.73	123
64884	D39	4/3/14	26.12	0	6.75	0.22	0.01	0.24	7.73	123
