# Supplementary material for: Distributions and Abundances of Sublineages of the N2-Fixing Cyanobacterium Candidatus Atelocyanobacterium thalassa (UCYN-A) in the New Caledonian Coral Lagoon
Source: Front Microbiol. 2018 Apr 5;9:554. doi: 10.3389/fmicb.2018.00554 (PMC5895702; doi:10.3389/fmicb.2018.00554)
Supplement: Supplementary file 2 [file Table_2.pdf]

Table 2: Discriminant Function Analysis (DFA) model to determine how environmental conditions varied across stations (e.g. if the station is predictable based on a set of environmental conditions) – Scoring Coefficient and Canonical Structure Table. Values indicate how environmental factors discriminate between stations.

Source	Scoring Coefficients: Canon 1	Total Canonical Structure: Canon 1	Scoring Coefficients: Canon 2	Total Canonical Structure: Canon 2
ln(temp)	-2.01846	0.1924225	2.9775578	-0.080746
ln(chl *a*)	1.4942655	0.4875073	2.8949591	0.508363
ln(silicate)	3.4836746	0.8626549	-1.032289	-0.227086
ln(NOx)	-0.347989	-0.28449	-0.16296	-0.045744
ln(SRP)	0.4431259	-0.138644	1.1749781	0.0923449
weekly ave precipitation	-0.1258	0.0074404	-0.082706	-0.032349
weekly ave wind speed	-0.387207	-0.024881	-0.127863	-0.01403
weekly ave wind direction	0.0117307	0.0125998	-0.003367	-0.057657
monthly ave precipitation	-0.465602	0.0959725	-0.103149	-0.164974
monthly ave wind speed	0.4662937	0.0403427	0.1703088	-0.052843
montly ave wind direction	-0.021061	-0.028406	-0.000887	-0.025671
nstar	0.3682769	0.1322696	0.9841624	0.2354044
