# Supplementary material for: Distributions and Abundances of Sublineages of the N2-Fixing Cyanobacterium Candidatus Atelocyanobacterium thalassa (UCYN-A) in the New Caledonian Coral Lagoon
Source: Front Microbiol. 2018 Apr 5;9:554. doi: 10.3389/fmicb.2018.00554 (PMC5895702; doi:10.3389/fmicb.2018.00554)
Supplement: Supplementary file 3 [file Table_3.PDF]

Supplementary Table 3: Quantitative (qPCR) abundances of UCYN-A1 and UCYN-A2 and the ratio of UCYN-A1 to UCYN-A2 abundance.

Sample	Date	Station	UCYN-A1 (*nifH* copies L^{-1})		UCYN-A2 (*nifH* copies L^{-1})		Ratio of A1:A2
			Average	Standard deviation	Average	Standard deviation	
64813	7/4/12	L2	5.16E+04	4.56E+03	7.09E+02	1.86E+02	27.59
64814	7/4/12	M09	5.06E+04	1.45E+03	1.23E+03	0.00E+00	27.65
64815	7/4/12	M99	1.33E+03	5.44E+01	4.74E+02	8.66E+00	6.62
64816	7/4/12	D39	2.67E+02	na	6.85E+02	7.36E+01	8.32
64817	8/3/12	L2	6.36E+04	9.46E+03	1.05E+03	4.68E+02	31.49
64818	8/3/12	M09	1.00E+00	na	1.67E+01	na	0.04
64819	8/3/12	M99	1.00E+00	na	1.67E+01	na	0.04
64820	8/3/12	D39	3.42E+03	1.69E+02	1.35E+03	3.43E+02	5.70
64821	10/2/12	L2	5.71E+05	7.36E+04	8.47E+03	4.56E+02	42.61
64822	10/2/12	M09	1.86E+03	6.01E+01	1.11E+03	2.15E+02	6.06
64823	10/2/12	M99	8.75E+03	8.17E+01	1.24E+04	8.79E+02	7.81
64824	10/2/12	D39	3.34E+03	5.69E+02	8.13E+02	3.36E+00	6.90
64825	11/27/12	L2	4.96E+04	6.68E+01	1.87E+03	1.50E+02	33.74
64826	11/27/12	M09	1.17E+05	2.74E+04	4.07E+02	1.72E+01	46.80

64827	11/27/12	M99	1.09E+04	4.03E+03	2.32E+03	4.22E+02	8.65
64828	11/27/12	D39	5.49E+03	6.74E+02	3.40E+01	na	5.63
64829	12/6/12	L2	2.24E+05	1.44E+04	1.83E+03	5.56E+01	26.45
64830	12/6/12	M09	4.83E+03	1.21E+01	2.27E+01	na	5.05
64831	12/6/12	M99	1.09E+05	9.25E+03	1.33E+03	7.58E+01	46.98
64832	12/6/12	D39	1.00E+00	na	2.43E+01	na	0.04
64833	1/31/13	L2	5.04E+02	2.56E+02	2.33E+01	na	12.86
64834	1/31/13	M09	2.17E+01	na	9.76E+02	5.95E+01	0.93
64835	1/31/13	M99	1.00E+00	na	3.13E+01	na	0.05
64836	1/31/13	D39	1.00E+00	na	1.00E+00	na	0.05
64837	2/28/13	L2	1.06E+04	1.59E+03	2.22E+01	na	8.62
64838	2/28/13	M09	2.27E+01	na	2.01E+02	6.04E+01	0.97
64839	2/28/13	M99	1.00E+00	na	3.51E+01	na	0.05
64840	2/28/13	D39	1.00E+00	na	3.92E+01	na	0.05
64841	3/27/13	L2	1.00E+00	na	1.00E+00	na	0.05
64842	3/27/13	M09	1.00E+00	na	2.23E+01	na	0.05
64843	3/27/13	M99	7.66E+02	6.03E+01	4.48E+01	na	19.01
64844	3/27/13	D39	3.57E+01	na	3.57E+01	na	1.34
64845	5/2/13	L2	4.11E+02	9.07E+01	2.50E+01	na	11.51

64846	5/2/13	M09	2.33E+01	na	2.33E+01	na	0.96
64847	5/2/13	M99	1.00E+00	na	1.00E+00	na	0.05
64848	5/2/13	D39	1.00E+00	na	1.00E+00	na	0.05
64849	6/12/13	L2	1.26E+05	2.99E+03	1.26E+03	3.08E+02	22.07
64850	6/12/13	M09	3.04E+02	8.80E+00	1.00E+00	na	8.94
64851	6/12/13	M99	7.41E+03	8.15E+02	2.22E+01	na	6.68
64852	6/12/13	D39	2.20E+03	3.02E+01	2.16E+01	na	5.25
64853	8/13/13	L2	2.12E+05	9.56E+02	2.25E+03	5.90E+01	26.08
64854	8/13/13	M09	1.33E+05	7.06E+03	2.02E+03	4.05E+02	18.97
64855	8/13/13	M99	8.17E+04	6.01E+03	7.01E+03	7.33E+02	36.31
64856	8/13/13	D39	9.97E+03	3.50E+02	1.47E+03	5.07E+02	8.90
64857	9/3/13	L2	4.36E+05	5.54E+04	8.13E+03	1.13E+03	33.28
64858	9/3/13	M09	3.74E+05	1.63E+04	5.71E+03	2.42E+02	30.16
64859	9/3/13	M99	4.84E+04	2.59E+03	2.50E+03	4.19E+02	35.85
64860	9/3/13	D39	3.14E+04	3.13E+03	1.34E+04	1.74E+03	23.61
64861	10/25/13	L2	4.46E+03	1.20E+02	2.13E+01	na	6.29
64862	10/25/13	M09	4.44E+03	1.28E+02	2.13E+01	na	6.48
64863	10/25/13	M99	2.34E+02	5.60E+00	3.07E+02	6.18E+01	7.48
64864	10/25/13	D39	1.00E+00	na	4.32E+02	2.48E+01	0.06

64865	11/25/13	L2	4.67E+03	4.20E+02	2.17E+01	na	5.74
64866	11/25/13	M09	2.51E+03	7.99E+01	2.13E+01	na	5.81
64867	11/25/13	M99	1.92E+03	2.46E+02	2.17E+01	na	4.72
64868	11/25/13	D39	1.99E+02	4.30E+01	2.27E+01	na	6.57
64869	12/17/13	L2	4.05E+02	6.15E+01	4.84E+02	6.47E+00	11.34
64870	12/17/13	M09	1.00E+00	na	2.50E+01	na	0.06
64871	12/17/13	M99	2.66E+01	na	2.66E+01	na	1.06
64872	12/17/13	D39	4.03E+01	na	4.03E+01	na	1.49
64873	1/22/14	L2	6.44E+03	2.82E+02	9.56E+02	1.21E+02	6.13
64875	1/22/14	M99	3.35E+02	6.65E+01	2.70E+01	na	9.54
64876	1/22/14	D39	3.57E+01	na	3.57E+01	na	1.43
64877	2/22/14	L2	2.60E+03	5.38E+01	1.31E+04	7.67E+02	5.49
64878	2/22/14	M09	1.04E+03	5.34E+02	3.63E+03	1.81E+02	23.21
64879	2/22/14	M99	1.00E+00	na	3.21E+01	na	1.00
64880	2/22/14	D39	1.00E+00	na	3.03E+01	na	1.00
64881	4/3/14	L2	1.24E+05	1.16E+04	1.12E+03	2.51E+01	34.16
64882	4/3/14	M09	1.00E+00	na	1.12E+03	2.57E+02	1.00
64883	4/3/14	M99	1.00E+00	na	6.00E+02	1.59E+02	1.00
64884	4/3/14	D39	1.00E+00	na	4.19E+02	5.20E+01	1.00
