# Supplementary material for: Distributions and Abundances of Sublineages of the N2-Fixing Cyanobacterium Candidatus Atelocyanobacterium thalassa (UCYN-A) in the New Caledonian Coral Lagoon
Source: Front Microbiol. 2018 Apr 5;9:554. doi: 10.3389/fmicb.2018.00554 (PMC5895702; doi:10.3389/fmicb.2018.00554)
Supplement: Supplementary file 4 [file Table_4.PDF]

Supplementary Table 4: Relative abundances of the top 12 oligotypes, listed in order of descending relative abundance. Oligotype sublineage is indicated in brackets.

Sample	oligo3 (A2)	oligo1 (A1)	oligo43 (A2)	oligo4 (A4)	oligo45 (A1)	oligo46 (A2)	oligo2 (A3)	oligo40 (A2)	oligo13 (A1)	oligo30 (A2)	oligo34 (A2)	oligo37 (A2)
64833	47%	9%	38%	0%	1%	0%	0%	0%	0%	0%	0%	0%
64834	50%	1%	43%	0%	0%	0%	1%	0%	0%	0%	0%	0%
64835	67%	0%	26%	0%	0%	0%	0%	0%	0%	1%	0%	0%
64836	83%	7%	0%	0%	0%	0%	0%	0%	0%	0%	0%	0%
64841	58%	17%	17%	0%	2%	1%	0%	0%	1%	0%	0%	0%
64842	81%	3%	10%	0%	0%	0%	0%	0%	0%	0%	3%	0%
64843	49%	18%	18%	4%	1%	1%	0%	0%	0%	0%	0%	1%
64844	60%	1%	36%	0%	0%	0%	0%	0%	0%	1%	0%	0%
64849	16%	68%	0%	0%	1%	2%	6%	1%	0%	0%	0%	0%
64850	77%	10%	6%	0%	1%	1%	0%	0%	0%	0%	0%	0%
64851	41%	44%	1%	0%	2%	2%	3%	1%	1%	0%	0%	0%
64852	32%	56%	0%	0%	2%	2%	0%	1%	0%	0%	0%	0%
64853	9%	88%	0%	0%	0%	0%	0%	0%	0%	0%	0%	0%
64854	17%	80%	0%	0%	0%	0%	0%	0%	0%	0%	0%	0%
64855	42%	46%	4%	0%	1%	1%	0%	0%	0%	0%	0%	0%
64856	52%	25%	20%	0%	0%	0%	0%	0%	0%	0%	0%	0%
64857	34%	54%	1%	0%	3%	4%	0%	1%	1%	0%	0%	0%
64858	33%	52%	4%	0%	3%	3%	0%	1%	1%	0%	0%	0%

64859	28%	39%	20%	5%	2%	1%	0%	0%	1%	0%	0%	0%
64860	39%	21%	29%	5%	1%	1%	0%	0%	0%	0%	0%	0%
64869	33%	19%	44%	0%	1%	0%	0%	0%	0%	0%	0%	0%
64870	41%	5%	50%	0%	0%	0%	0%	0%	0%	1%	0%	0%
64871	30%	17%	36%	14%	0%	0%	0%	0%	0%	0%	0%	0%
64872	35%	19%	20%	24%	0%	0%	0%	0%	0%	0%	0%	0%
64881	8%	78%	0%	0%	1%	1%	4%	0%	0%	0%	0%	0%
64882	92%	0%	4%	0%	0%	0%	0%	0%	0%	0%	0%	0%
64883	97%	0%	0%	0%	0%	0%	0%	0%	0%	0%	0%	0%
64884	88%	4%	5%	0%	0%	0%	0%	0%	0%	0%	0%	0%
