# Supplementary material for: Distributions and Abundances of Sublineages of the N2-Fixing Cyanobacterium Candidatus Atelocyanobacterium thalassa (UCYN-A) in the New Caledonian Coral Lagoon
Source: Front Microbiol. 2018 Apr 5;9:554. doi: 10.3389/fmicb.2018.00554 (PMC5895702; doi:10.3389/fmicb.2018.00554)
Supplement: Supplementary file 6 [file Image_2.pdf]

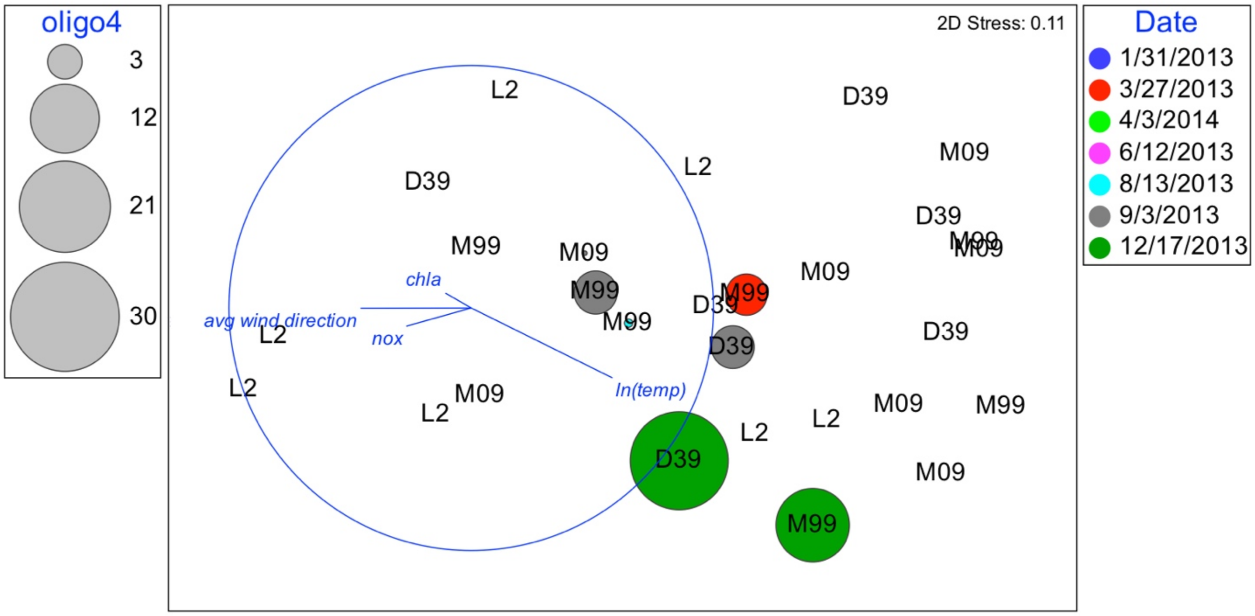

Supplementary Figure 2: Nonmetric Multidimensional scaling (NMDS): plot of 28 biological samples, coded by date and station with best explanatory environmental variable overlay. Relative abundances of oligo4 are represented by the diameter of the circles.
