# Supplementary material for: Distributions and Abundances of Sublineages of the N2-Fixing Cyanobacterium Candidatus Atelocyanobacterium thalassa (UCYN-A) in the New Caledonian Coral Lagoon
Source: Front Microbiol. 2018 Apr 5;9:554. doi: 10.3389/fmicb.2018.00554 (PMC5895702; doi:10.3389/fmicb.2018.00554)
Supplement: Supplementary file 7 [file Image_3.PDF]

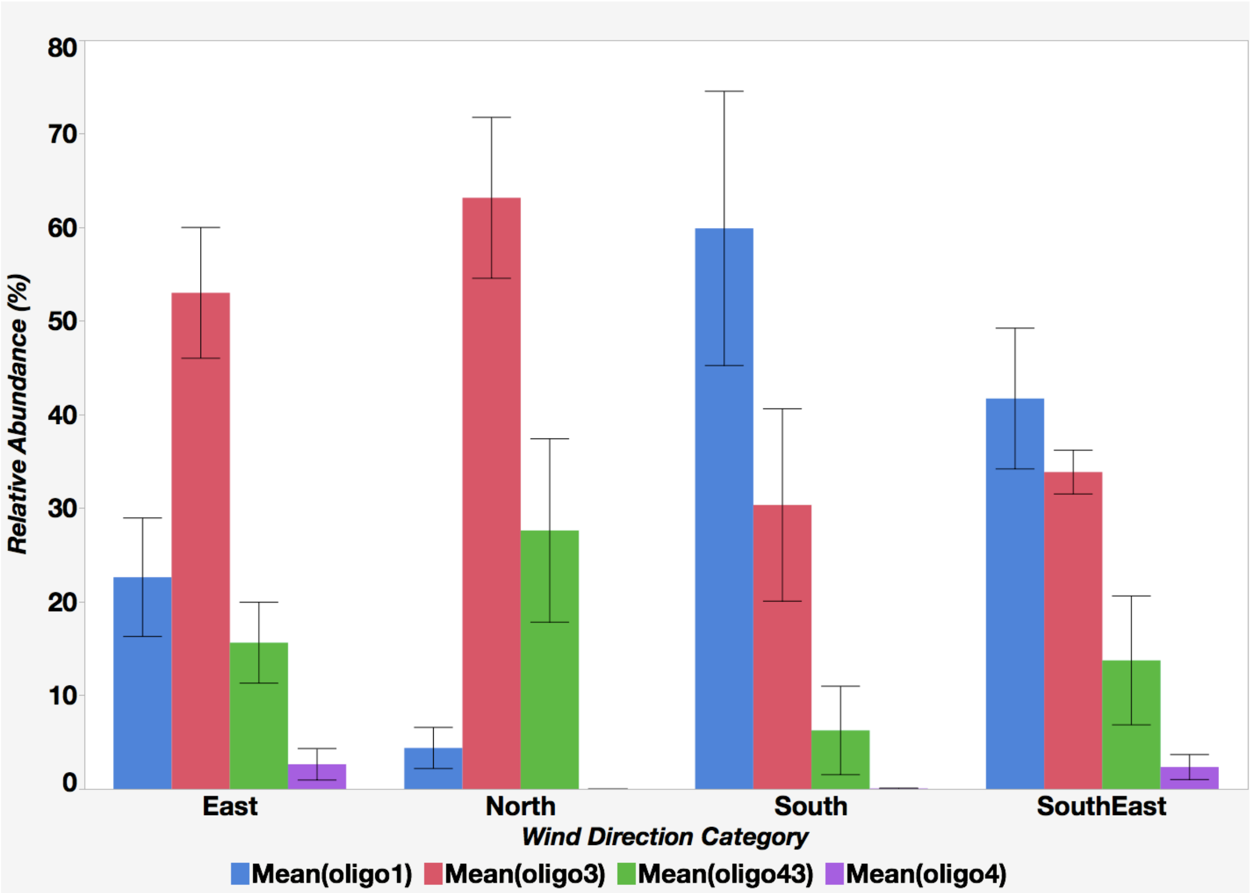

Supplementary Figure 3: The relationship between mean oligotype relative abundance and weekly average wind direction category. Categories are defined as follows: north (340°-20°), east (80°-120°), southeast (121°-160°), and south (161°-110°).
